# Health Surveillance Response to the Oropouche Outbreak in Alfredo Chaves, Espírito Santo, Brazil

**DOI:** 10.1590/0037-8682-0345-2025

**Published:** 2026-02-16

**Authors:** Cintia Lepaus Thomas, Karllian Kerlen Simonelli Soares, João Paulo Cola, Thiago Nascimento do Prado, Ethel Leonor Noia Maciel

**Affiliations:** 1 Prefeitura Municipal de Alfredo Chaves, Secretaria de Vigilância em Saúde, Vitória, ES, Brasil.; 2 Universidade Federal do Espírito Santo, Vitória, ES, Brasil.; 3 Secretaria de Estado da Saúde de Espírito Santo, Vitória, ES, Brasil.

This text summarizes the health surveillance actions and coordination efforts taken to control the Oropouche outbreak in Alfredo Chaves, Espírito Santo, Brazil, from September 2024 to February 2025. Oropouche fever (OF) is an emerging zoonosis caused by the Oropouche virus (OROV), an arbovirus of the genus Orthobunyavirus and the family Peribunyaviridae. It was first discovered in 1955 in Vega de Oropouche in Trinidad and Tobago. In Brazil, it was isolated in 1960 from a sloth and isolated cases and outbreaks occurred in the Amazon region^1^.

The main mode of transmission is through the bites of Culicoides paraens mosquitoes. These insects live in forests and rural areas with monoculture cultivation, especially in banana, as well as in peri-urban zones. Females lay their eggs in moist organic matter, such as fallen fruit peels, foliage, stream banks, and decaying tree trunks. In banana plantations, reproduction occurs at the base of the banana tree stem^2^.

The symptoms of oropouche fever are similar to those of dengue fever and usually last from two to seven days, with recurrence in 60% of cases after one to two weeks. It is important to highlight the risk of the vertical transmission of OROV, which can cause severe fetal damage, microcephaly, and even death^1^.

According to a study by Fiocruz, the new lineage of the virus that emerged between 2010 and 2014 in the state of Amazonas was responsible for the recent epidemic (2022-2024) in several Brazilian states^3^. The number of confirmed FO cases in Brazil between 2024 and 2025 (April) is alarming, totaling 22,955 (HEALTH, 2025). Of this, the state of Espírito Santo contributed to more than half of the cases (51.5%; 11,826 notifications), with the municipality of Alfredo Chaves being one of the most affected by the disease, with 1, 289 confirmed cases in the same period.

Given this situation and considering the challenges posed by the emergence of a new disease in a population of susceptible individuals, characterized by the initial under-reporting of cases due to their clinical similarity to dengue and the limited local diagnostic capacity to differentiate these arboviral infections, together with the absence of clinical protocols, the progressive increase in the number of cases, the high density of the main vector in the municipality, and the logistical difficulties of conducting surveillance in a small municipality with an extensive predominantly agricultural rural area (especially banana cultivation), important weaknesses in local preparedness and response capacity were evident. Therefore, this report aimed to describe the Health Surveillance actions implemented in response to the outbreak, highlighting the strategies adopted and results achieved.

This study was conducted in the municipality of Alfredo Chaves, located in the southern part of the state of Espírito Santo. The municipality has a large rural area, and its main economic activities include banana cultivation and livestock farming. Alfredo Chaves covers 615 km², is located 81 km from the state capital, Vitória, and has a population of 13,8367. The climate is hot and subtropical, with intense rainfall from October to November. It is a mountainous area with an irregular terrain. These characteristics may favor the reproduction and persistence of vectors. For example, Culicoides paraensis breeds in organic matter typical of rural areas. The predominance of agriculture and fruit cultivation (particularly banana farming) in Alfredo Chaves may explain the high density of these vectors, as such environments provide suitable oviposition sites for female mosquitoes.

In Espírito Santo, cases were notified through the e-SUS Health Surveillance (e-SUS VS), a computerized system implemented by the State Health Department, to register, consolidate, and monitor events of interest in public health. This platform allows for the completion of notification forms, monitoring of epidemiological investigations, and integration of data between municipalities and the state, functioning as the official information base for analysis and decision-making in the Espírito Santo territory. Municipal bulletins, meeting minutes, field reports, and laboratory records were also used.

The expansion of testing to Oropouche was one of the main strategies, especially in cases previously diagnosed with dengue, Zika, or chikungunya. With the confirmation of the first autochthonous cases in Espírito Santo, the state laboratory (Lacen) began testing all samples that tested negative for arboviruses and OROV, thereby increasing case detection in the region. In September 2024, during epidemiological weeks 36 to 38, two cases of acute febrile illness with symptoms similar to dengue were identified but presented negative results in rapid tests. The Epidemiological Surveillance team initiated an active investigation and sent samples to Lacen-ES. In one month, 62 cases were confirmed by RT-PCR, triggering an emergency response. The peak of notifications and confirmations occurred in epidemiological week 43, with 40 cases between October 20 and 26, and 647 confirmed cases by November. The cases were prevalent in women aged over 41 years residing in urban areas. However, this is a municipality characterized by peri-urban environments.

Given this scenario, Health Surveillance integrates theoretical and practical knowledge essential for a coordinated response, uniting the actions of epidemiological surveillance, environmental surveillance, Primary Care, and the laboratory.

Thus, an intersectoral response structure was created, and the Health Surveillance Coordination convened the Municipal Arbovirus Control Committee. The responses involved representatives from Epidemiological, Environmental and Health Education fields. Standardized protocols were created and implemented for case investigations, vector control, and laboratory surveillance. One of the health units became a point of reference, with a doctor caring exclusively for patients with compatible signs and symptoms. Therefore, immediately after consultation, the patient was referred for blood collection for testing, preventing them from traveling to a clinical analysis laboratory. Hydration rooms were implemented to serve residents who required greater care more quickly.

Furthermore, epidemiological and environmental investigations were conducted during home visits to identify secondary cases, collect data, monitor contacts, identify likely locations of infection, and understand the magnitude of the local situation.

To reduce vector density, environmental surveillance inspected vacant lots and high-risk areas and issued notifications to owners for cleaning and mechanical management of the environment. These actions occurred mainly in the urban area of the municipality because, in rural regions, where banana planting is the main agricultural activity, with little possibility of mechanical management, the guidance was the usual protection through a physical barrier to bites (long clothing, use of repellents, and mineral oils).

Some of the most notable aspects were risk communication and community mobilization. Health Education teams created accessible materials about the disease, its prevention, and necessary care. Dissemination efforts expanded through local radio stations, social media platforms, and schools. Health agents, community leaders, and churches participated in the clean-up and door-to-door awareness campaigns.

Ninety-two cases were confirmed using RT-PCR between January and February 2025. The community has largely accepted these measures. A significant drop in incidence occurred after the first three weeks of the response, as shown in Figure 1. However, the observed decrease in cases may be related to a reduction in the number of susceptible individuals exposed to the viral circulation.

**FIGURE 1:**
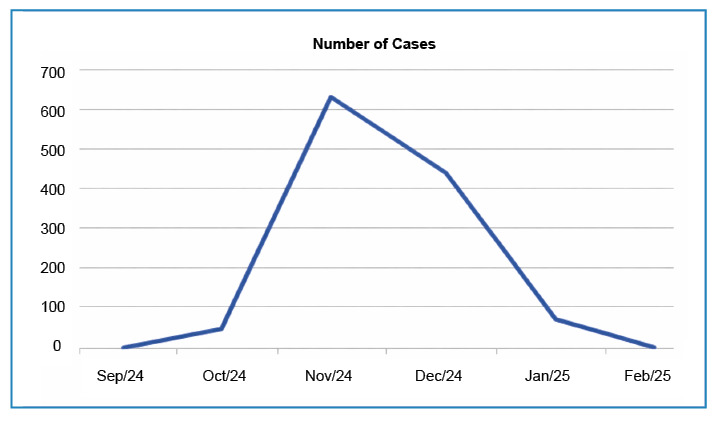
Graphical representation of Oropouche fever cases reported in Alfredo Chaves, Espírito Santo, Brazil, from 2024 to 2025. **Source:** Alfredo Chaves Municipal Health Department.

## FINAL CONSIDERATIONS

The experience in Alfredo Chaves demonstrated a response arrangement that can be adapted to other small municipalities with extensive rural areas and strong agricultural production, provided they have the minimum conditions for organizing Health Surveillance. Decentralization of sample collection points, systematic and timely use of health information systems, integration of epidemiological and environmental surveillance and primary healthcare, and intersectoral coordination are replicable components of the adopted model.

More than simply following a trend observed in other settings, the experience of Alfredo Chaves illustrates how municipalities with similar characteristics can organize faster and more effective responses to the emergence of the Oropouche fever. For its replication, consolidated information systems, a functioning laboratory network capable of confirming cases in a timely manner, and active intersectoral coordination bodies that integrate health, agriculture, and other strategic sectors are essential. When articulated, these elements enhance the local capacity to detect outbreaks early and implement control measures in rural areas with high vector density.

Health surveillance played a key role in controlling the Oropouche outbreak in Alfredo Chaves. This experience highlights the importance of vigilant surveillance, the integration of technical areas, and communication with the community. This emblematic case of resilience and rapid epidemiological response was fostered by collaboration between scientific institutions at the municipal, state, and federal levels.

It is recommended that local contingency plans be maintained, continuous training offered, and laboratory networks strengthened to respond to emerging arboviruses. Vector control interventions also need to be improved and are already being addressed through partnerships with the Ministry of Health and Fiocruz.

Experimental studies are being conducted in Alfredo Chaves, including the use of insecticides already employed in *Aedes sp*. control, which have shown promising results in reducing the density of *Culicoides paraensis*, as well as in partnership with EMBRAPA to develop banana crop management strategies aimed at vector reduction.

Climate change has directly affected the dynamics of several infectious diseases such as Oropouche fever and other arboviruses. Agricultural production, environmental conditions, deforestation, and other human activities create environments favorable for the proliferation of vectors, such as mosquitoes and midges. As a result, previously non-endemic areas are now experiencing outbreaks, which is expanding the geographic distribution of these diseases, and posing increasing challenges to surveillance and public health systems. The link between climate and health highlights the urgency for integrated actions that consider both epidemiological control and adaptation to environmental changes.

## Data Availability

Research data is only available upon request
